# Reliability of upright posture measurements in primary school children

**DOI:** 10.1186/1471-2474-6-35

**Published:** 2005-06-29

**Authors:** Maureen P McEvoy, Karen Grimmer

**Affiliations:** 1 School of Health Sciences, University of South Australia, North Tce., Adelaide, 5000, Australia; 2 Centre of Allied Health Evidence, collaborating centre of the Joanna Briggs Institute, GPO Box 2471, Division of Health Sciences, University of South Australia, North Tce., Adelaide, 5000, Australia

## Abstract

**Background:**

Correct upright posture is considered to be a measure of good musculoskeletal health. Little is known about the usual variability of children's upright standing posture. The aim of this study was to assess differences between repeated measures of upright posture in a group of primary school children.

**Methods:**

Sagittal plane photographs of usual, relaxed upright standing posture of 38 boys and girls aged 5–12 years were taken twice within an hour. Reflective markers were placed over the canthus, tragus, C7 spinous process, greater trochanter and lateral malleolus. Digitising software was used to calculate the x,y plane coordinates, from which five postural angles were calculated (trunk, neck, gaze, head on neck, lower limb). Height, weight, motor control estimates (as measured by the Brace Tests) and presence of recent pain were recorded for each child, and the association between the first test measure of posture angles and these factors was assessed using linear regression and ANOVA models. Multiple ANOVA models were applied to analyse the effect of repeated testing, and significant predictors on the angles.

**Results:**

Four of the five postural angles (trunk, neck, head on neck, lower limb) were significantly influenced by age. As age was strongly associated with height (r^2 ^= 0.84) and moderately associated with weight and motor control (r^2 ^= 0.67, 0.56 respectively), these developmental parameters may well explain the age effect on angles. There was no relationship between age and pain reported on either the testing day, or recently, and there was no gender influence on any angle. There was no significant effect of repeated testing on any angle (ICC>0.93). None of the hypothesized predictors were associated with differences in angles from repeated testing.

**Conclusion:**

This study outlined the variability of relaxed upright standing posture of children aged 5–12 years, when measured twice in an hour. Age influenced the size of the angles but not the variability. While the subject numbers in this study are small, the findings provide useful information on which further studies in posture and its development in pre-adolescent children can be based.

## Background

Posture reflects the relationship between spinal segments, and the influence of the environment on spinal segments [1]. Correct upright posture is considered to be an important indicator of musculoskeletal health. Costs associated with musculoskeletal impairments in health and loss of work, have contributed to a growing interest in optimizing posture, particularly in relation to sitting positions associated with the use of visual display units [2] and standing posture in children in relation to backpack use [3].

There is no standard approach to measuring posture. Photographic observations of ideal posture have been ranked visually or simple equipment such as a tape measure, penciled landmarks and a plumbline, have been used [4,5]. The linking of body landmarks has given angular measurements, allowing a more quantitative assessment of posture [6]. Watson and Mac Donncha [6] reported 85% reliability when ten aspects of adolescent photographic posture were qualitatively categorized and rated. Straker and Mekhora [2] photographically evaluated sitting postures as a series of angles in adults working at visual screens. This method was reported in Straker et al [7] to have previously shown reliability (r^2^>0.8) in adults. Grimmer et al [8] adapted the measurements used by Straker and Mekhora [2] to assess standing posture in adolescent high school students aged 12–18 years. The reliability of this photographic method of posture assessment however has not been tested in children. Indeed the measurement of posture in children has received scant attention in the literature and little is known about the variability of children's standing posture.

There are many factors which may influence the reliability of photographic posture assessment in children. These include maturation and developmental factors such as age, gender, height and the development of postural control and co-ordination. The presence of pain and the testing environment may also have an effect. Factors associated with the measurement process including palpation of bony landmarks for marker placement and reproducibility of the digitization process, may also contribute to the reliability.

Growth spurts occurring in 9–12 year olds may cause widespread alterations in body shape and dimensions and have an effect on muscle tightness and flexibility, all of which may influence posture in children [9-11]. Children have relatively larger heads and also a higher center of mass at about T12, compared to L5-S1 in adults. The combination of being shorter and having a higher center of mass may result in increased sway in children and difficulty in maintaining static balance [1]. The stage of development of postural responses may influence the ability of the child to maintain a relaxed standing posture. Postural control development occurs sequentially in a cephalo-caudad direction with head control first, followed by the trunk and then postural stability in standing [1]. The motor and sensory systems involved in postural stability go through a transition period at 4–6 years and reach adult maturity by 7–10 years [12-14].

Spinal pain is recognized as a significant influence on normal posture in adults, with an antalgic posture commonly taken up to avoid a painful position [15-17]. It has been proposed that pain may indirectly affect posture, through the alteration of somatosensory signals to the central nervous system [18]. There is no reason to suspect that this effect would be different for children.

The aim of this study was to assess the variability of children's posture using repeated measures within the same hour.

## Methods

### Study design

A repeated measures observational study was conducted in December 2000. This study formed part of a larger epidemiological study conducted by the Centre for Allied Health Evidence, University of South Australia, where primary school students participated in a range of tests, to identify factors which may contribute to spinal health. In addition to posture, other measures included anthropometric, school bag weight, muscle endurance, coordination and a questionnaire about spinal symptoms and activity levels. There were six separate testing stations at which children were measured, with time taken per station for testing ranging from three minutes (anthropometric) to 15 minutes (muscle endurance).

### Sample selection

One class from each year level (Reception, aged 5 years to Year 7, aged 12 years) of a large suburban Adelaide primary school was chosen to be involved in the larger study. Ethical approval was gained from the Ethics Committee of the University of South Australia and the Department of Education and Children's Services, and written parental consent was obtained prior to the commencement of the study. All consenting children were included. Children arrived at the testing area in class level groups of varying sizes. As only a short time was allotted by the school for testing, the measurements at each test station were determined by the order that children approached the six test stations, the speed of testing at each station and the availability of places at the next test station. Before the arrival of the subsequent class group, children who had completed all the testing stations once, were re-tested, forming a sample of convenience for the posture reliability study.

### Equipment, preparation and testing procedure

Subjects were tested in the school gymnasium and efforts were made to control for temperature, noise and distractions. In an attempt to minimise data collection error, research assistants at all stations received comprehensive training in the use of study test protocols prior to commencement of the study. Strict protocols were used to ensure the correct placement of anatomical markers, positioning of the subject and camera placement [8].

**Figure 1:**
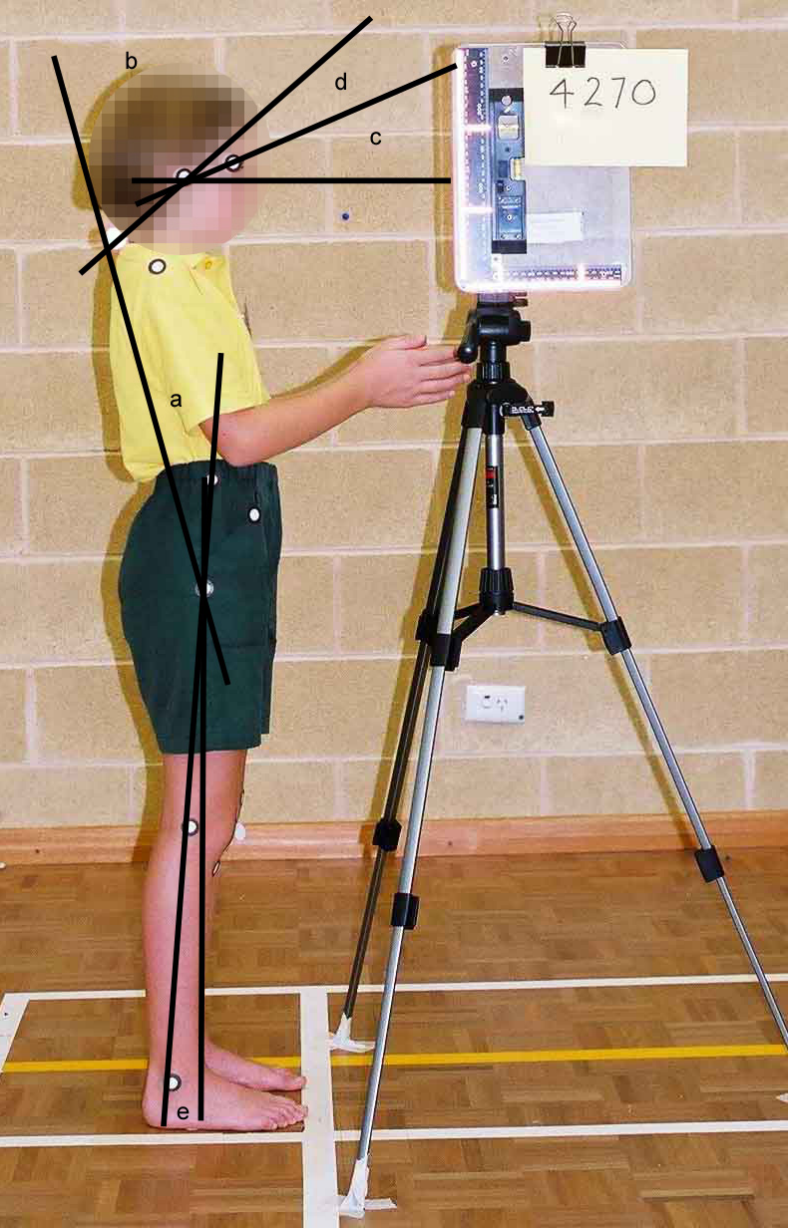
Adhesive marker placement and postural angles. a trunk angle; b neck angle; c gaze angle; d head on neck angle; e lower limb angle

To capture postural information on body segments, adhesive markers were placed over right-sided lateral landmarks including the lateral canthus of the eye, the tragus, the greater trochanter and the lateral malleolus. A small reflective ball was placed over the spinous process of C7, to ensure that this landmark would be detected on the scanned photographs (Figure 1). The markers that were placed over the shoulder, pelvis and knee were not included in the angle calculations used for this study, however they were used for other research purposes [8]. As the study aimed to assess variability in posture on repeated occasions of testing, the markers were left in place between tests. Removing and then replacing the markers would have introduced an additional element of reliability of the examiner in marker placement, which was not the aim of this investigation.

Portrait – format photographs were obtained using a Canon SLR camera (EOS 500) that was attached to a tripod and placed at a distance of 3.1 m and in a direct line from the subject. Spirit level adjustments placed on the top of the camera and the front of the lens confirmed horizontal and vertical alignments of the camera respectively. The tripod was secured in the correct position on the floor using masking tape. Floor markers were used to standardise subject placement and to ensure that the subject's right side was aligned perpendicular to the camera. A set-square and spirit level, attached to a perspex calibration board, were connected to a second tripod. The calibration board was placed in the field of view and aligned with the subject to allow referencing of horizontal and vertical axes from the photographs. The calibration board also displayed each subject's identification number.

For positioning, the child was instructed to stand comfortably in a normal standing position 'as if waiting at the canteen', and to look straight ahead at a pre-determined point on the wall. To allow for visualization of the greater trochanter marker, the subject was further instructed to move the elbows forward but still touching the body and with minimal shoulder movement. The position was checked by a researcher and by the photographer prior to the photograph being taken. After the first photograph, the subject was asked to move away from the testing station, walk around a small area and then return to the photographic position where the second photograph was taken. The anatomical markers were not moved between photographs, and their position was rechecked prior to the second photograph to ensure that they were securely in place.

The letter 'R' was recorded after the subject identification number to indicate the 'repeat' photographs. This method of posture measurement is reported elsewhere by Grimmer et al [19].

Measurement of factors which potentially influenced posture was restricted to age, gender, height, weight, recent pain and gross motor control ability in relation to co-ordination, strength, balance and flexibility (using the Brace test) [20]. The Brace tests are validated gross motor skill tests that vary in complexity from very simple (eg walk heel to toe for 10 steps along a line) to very difficult (eg with the free foot, jump over the hand holding the opposite foot). The subject received a score of one (success) or zero (failure) for each test to a maximum total score of 20. Self-reported outcomes of age, gender and recent pain were obtained from a parent or carer, in conjunction with the child, and recorded on the study questionnaire. If pain was present on the day of testing or had occurred during the previous week, this was recorded by ticking boxes that corresponded to the correct body part (eg head, neck, upper back, elbow or knee). The pain variable used in the statistical analysis for this paper reflected any pain reported in the neck, upper or lower back. The child's weight (in kilograms) and height (in centimetres) was measured in accordance with standard anthropometric protocols used in previous research conducted by our group [19].

### Digitising and synthesising posture data into angles

After testing was completed and films developed, a negative scanner (Nikon LS-2000) was used to convert developed negatives into electronic format files. Image analysis software (ImageTool UTHSCA Version 2.0, University of Texas Health Science Center, San Antonio, TX) was employed to digitise the x and y plane coordinates obtained from each anatomical landmark from the photographs. A strict pre-existing protocol for scanning and digitization was followed, which reduced repeated measure estimates of landmark coordinates from any photograph to less than 10 pixels difference (non-significant (p > 0.05)). One researcher undertook all scanning and digitizing to eliminate inter-examiner error.

The x, y coordinate values were imported into MS Excel spreadsheets for calculation of the five body angles used for data analysis (Figure 1).

#### Trunk Angle

The angle between the trunk (as indicated by a line drawn through anatomical markers at C7 and the greater trochanter) and a vertical line through the greater trochanter.

#### Head and Neck on Trunk Angle (abbreviated to "Neck Angle" for this article)

The angle between the head and neck segments, as indicated by a line drawn through anatomical markers at C7 and the tragus of the ear, and the trunk, as indicated by a line drawn through anatomical markers at C7 and the greater trochanter.

#### Gaze Angle

The angle formed by a line drawn through anatomical markers at the canthus of the eye and tragus of the ear and a horizontal line through the tragus of the ear.

#### Head on Neck Angle

The angle between the neck as indicated by a line drawn through anatomical markers at C7 and the tragus of the ear, and the head as indicated by a line drawn through the canthus of the eye and the tragus of the ear.

#### Lower Limb Angle

The angle between the lower limb as indicated by a line drawn through anatomical markers placed at the greater trochanter and the ankle, and the vertical, as indicated by a vertical line drawn through the greater trochanter.

Trunk, neck, gaze and head on neck angles were adapted from Straker et al [7] and Straker and Mekhora [2], who used a mid-iliac marker in place of the greater trochanter and a marker over the external auditory meatus in place of the tragus marker. Straker et al [7] and Straker and Mekhora [2] developed angles to assess sitting posture particularly in relation to use of visual display units. These angles were modified for the current study to minimize error of marker placement by using accessible and specific anatomical landmarks. A fifth angle, the lower limb angle, was established for standing posture assessment, for the purposes of this study.

### Testing and statistical analysis

**Figure 2:**
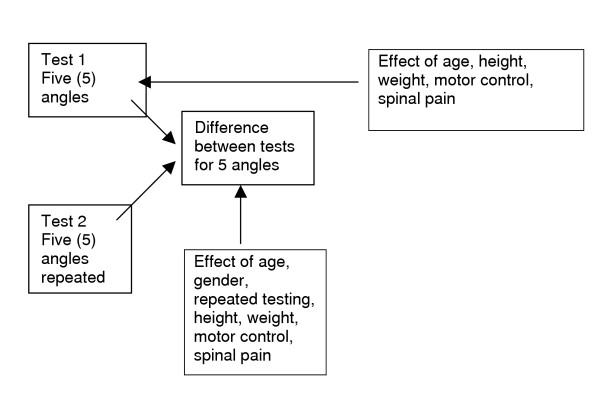
The testing approach

The testing approach is summarized in Figure 2.

Four age groups were constructed, these being less than or equal to 6 years, 7–9 years, 10–11 years and greater than 11 years. These divisions were determined on age quartiles in the data, which provided appropriate groupings to reflect structural and functional changes in children related to their growth and maturation [21].

The mean values and standard deviations (SD) for each angle in each age group for the first set of measurements (Test 1) were determined.

An understanding of the variability of the posture angles relative to the mean for the first set of measurements was determined for each age group, by dividing the standard deviation by the mean.

To understand what may influence posture, the association between the first set of posture measures and potential predictors of children's postures (height, weight, motor control, spinal pain) were individually assessed using univariate linear regression models, or in the case of spinal pain, an ANOVA model. A *p *value of < 0.01 was chosen to reduce the possibility of missing an important effect.

The strength of the linear association was determined using criteria by Dawson and Trapp [22], where r^2 ^<0.25 indicates a poor relationship, 0.25-0.5 indicates a fair relationship, 0.5–0.75 indicates a moderate to good relationship, r^2^>0.75 indicates a strong relationship.

The 95% confidence intervals (CI) were reported around the mean differences in angles within age groups, to provide information on usual variability of young people's posture for future studies. Confidence intervals that do not include zero indicate a statistically significant difference at the 5% level.

Intraclass correlation coefficients (ICC _1,3_) were used to identify the reliability between test and retest angle measure differences based on the within and between subject variances, obtained from output from the ANOVA models [23].

Multiple ANOVA models were used to examine the effects of potential predictors (age, gender, height, weight, motor control repeated testing and pain) on posture outcome measures. Scheffe's post hoc multiple comparisons test was used [23].

Post hoc power calculations were performed, using the effect sizes derived from this study to determine if the study sample had sufficient power to enable a true difference in estimates to be detected if they truly existed [23,24].

## Results

### Descriptive data

There were 38 primary school students in the sample, whose demographic details are outlined in Table 1. Mean (SD) for height, weight and motor control (Brace Test scores) are provided per age group in Table 2. The height and weight of the sample did not differ remarkably from normative data for same aged children in the western world [25].

**Table 1:** Gender of children by age group.

	< = 6 yrs (n = 9)	7–9 yrs (n = 9)	10–11 yrs (n = 12)	>11 yrs (n = 8)
Girls	3	3	7	2
Boys	6	6	5	6

**Table 2:** Mean (SD) posture predictors by age group.

	< = 6 years n = 9	7–9 years n = 9	10–11 years n = 12	>11 years n = 8
Height (cms)	112.1 (3.7)	130.0 (7.8)	138.8 (5.8)	146.9 (5.4)
Weight (kgs)	33.7 (9.2)	29.7 (7.2)	32.4 (3.8)	42.5 (6.2)
Brace tests (number out of possible 20)	13 (4.0)	10 (3.0)	14 (3.0)	13 (3.0)

On the day of testing four children (11%) reported spinal pain (two each in the youngest and oldest age groups) and 14 children (37%) reported experiencing spinal pain (2 each in the youngest and oldest age groups, 4 in the 7–9 year olds, and 6 in the 10–11 year olds) in the week prior to testing. There were no gender differences within any age group for any of these measures.

### Variability in angle measurements

**Figure 3:**
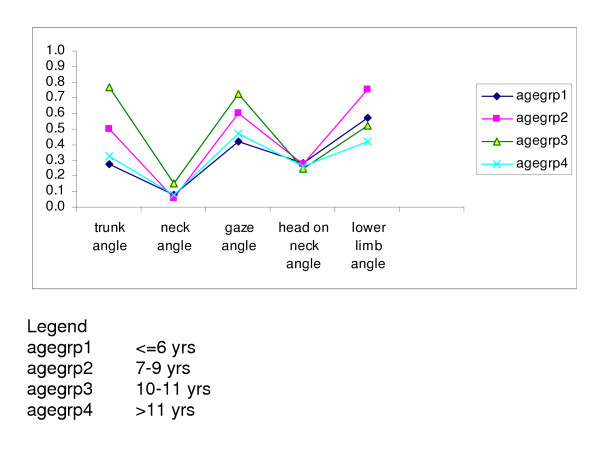
Relative variability of the first set of measurement of the angles for each age group

The mean (SD) angles (in degrees) for each of the five angles measured on Test 1 in each age group are presented in Table 3. Negative angles were found in 97% of the first set of trunk angles, 4% of the gaze angles and 20% of the lower limb angles. The variability of the posture angles relative to the mean in Test 1, is illustrated in Figure 3. Similar relative variability was found for the youngest and oldest age groups for all angles except the lower limb angle, which seemed the most variable over all the age groups. The age groups with the greatest relative variability were the 7–9 and 10–11 years groups, particularly for the trunk, gaze and lower limb angles. The least variable angles over all the age groups were the neck angle and the head on neck angle.

**Table 3:** Mean (SD) test 1 for each angle (in degrees) for each age group.

	<= 6 years	7–9 yrs	10–11 yrs	>11 years
Trunk angle	-8.8 (2.5)	-5.0 (2.6)	-5.0 (4.3)	-5.6 (2.6)
Neck angle	61.4 (5.3)	58.5 (3.4)	55.7 (8.7)	51.6 (4.9)
Gaze angle	12.3 (5.4)	11.1 (7.9)	10.3 (7.6)	13.1 (9.0)
Head on neck angle	25.1 (7.5)	25.5 (7.3)	29.1 (7.4)	30.9 (9.6)
Lower limb angle	3.3 (2.6)	0.05 (1.2)	2.1 (1.9)	2.9 (2.0)

### Examining predictors of posture angles

In this sample, age was strongly associated with height (r^2 ^= 0.84) and moderately associated with weight and motor control (r^2 ^= 0.67, 0.56 respectively). There was an unconvincing association between any of the postural angles, height, weight and motor control (see Table 4), with the strongest associations observed for the trunk angle with height, weight and motor control. Spinal pain was not associated with age, or any of the postural angles (p > 0.05).

**Table 4:** The effect of height, weight and motor control (Brace Tests) on the five body angles.

Angle	Height	Motor control	Weight
Trunk angle	0.22*	0.18*	0.22*
Neck angle	0.13	0.16	0.11
Gaze angle	0.02	0.003	0.004
Head on neck angle	0.03	0.01	0.004
Lower limb angle	0.06	0.03	0.003

(* indicates significant findings p < 0.01)

### Differences between tests

The mean differences (95%CI) (in degrees) between tests 1 and 2 for each angle in each age group are presented in Table 5. The ICC_1,3 _values for repeated measures of the five angles, ranged from 0.93 to 0.99, suggesting no significant effect of testing on any angle.

**Table 5:** Mean differences (95%CI) between test 1 and 2 for each angle (in degrees) by age group.

	< = 6 years n = 9	7–9 years n = 9	10–11 years n = 12	>11 years n = 8
Trunk angle diff	0.2 (-2.6 to 2.9)	1.7 (-0.8 to 4.1)	2.04 (-1.0 to 5.0)	1.3 (-1.4 to 4.0)
Neck angle diff	-0.5 (-5.7 to 4.8)	1.5 (-2.7 to 5.6)	-1.9 (-8.4 to 4.7)	-1.4 (-7.6 to 4.8)
Gaze angle diff	-2.2 (-7.2 to 2.8)	-2.5 (-9.5 to 4.5)	2.7 (-3.8 to 9.1)	2.5 (-5.3 to 10.3)
Head on neck angle diff	2.5 (-4.6 to 9.5)	-0.1 (-7.6 to 7.4)	-2.8 (-9.2 to 3.5)	-2.4 (-10.7 to 6.0)
Lower limb angle diff	-1.1 (-4.0 to 1.8)	-0.7 (-2.2 to 0.8)	-0.6 (-2.4 to 1.1)	-0.6 (-2.6 to 1.3)

### Examining predictors of differences between posture angles

Multivariate ANOVA models found no effect of gender on the mean test difference for any angle. However, there was a significant age effect (p < 0.01) on the difference between repeated measures of the trunk angle, the neck angle, the head on neck angle and the lower limb angle i.e. for all but the Gaze Angle. Height, weight, motor control and pain were found to have no influence on the difference between test -retest measurements (p > 0.05). Scheffe's post-hoc test [23] however, found that only three percent of the observations for repeated test differences between any angle were significant.

Post-hoc power calculation using the effect size directly derived from the results of the study [24] indicated that the sample was sufficiently powered at 0.80 and p < 0.05 for the trunk angle and the lower limb angle, to detect the effect of repeated testing. Greater numbers were required for 80% power (p < 0.05) to test the effect of repeated measures of the other three angles. Using a specific sample size electronic calculator [24], and based on the effect-size data from this study, 32% power for repeated testing of the neck angle was found (for 80% power, 146 subjects required), 25% power for repeated testing of the gaze angle (for 80% power, 360 subjects required) and 28% power for repeated testing of the head on neck angle (for 80% power, 312 subjects required).

## Discussion

This study provides rare information on the repeatability of relaxed standing posture in children aged 5–13 years, with posture expressed as five angles derived from x,y coordinates of anatomical points, calculated electronically from photographs. As the height and weight of our sample did not differ remarkably from normative data for same aged children in the western world [25] and as our sampling approach should not have incurred bias beyond chance, we believe that this paper presents useful externally generalisable information not only for clinical purposes, but also to inform further research on larger numbers of children, particularly to test reliability of neck angle, gaze angle and head on neck angle.

### Subject measurement

The students were tested in a limited time period under minimum stressed conditions (i.e. there was little opportunity for fatigue). They were moved to the testing stations in a variable order, as a position became available for testing, thus it is possible that the order of testing may have influenced the results. As testing was completed within a period of 40–60 minutes, fatigue or boredom bias is considered unlikely. The most likely reason for differences in measurements would be natural variability in subject responses to the test-retest situation [1]. As so little is known about young people's postural variability, a study with larger numbers is required to clarify this issue.

### The reliability of the posture measurements

This study suggested that children's standing posture (quantified by five whole body or segmental angles) did not change significantly on repeated testing. Further testing with larger subject numbers, is required however, to be certain of these findings for the neck, gaze and head on neck angles. The five posture angles are considered useful and easily attained postural outcome measures that may be appropriate for application to clinical studies. The trunk angle is a measure of the trunk position relative to the line of gravity. The negative values shown for the trunk angle in Table 3 indicate a relative backward lean of the trunk and this angle is much greater for the children 6 years and under. The digitized marker points on C7 and the greater trochanter are a long distance apart, thus the protocols which guided accurate marker placement and subject positioning were essential to minimize error in measurement. The mean neck angle across the age groups ranged between 61 and 51 degrees. This angle is a measure of the head and neck position in relation to the trunk and gives a measure of the forward head position, which is a useful clinical indicator of mid/lower cervical spine dysfunction. A lower measurement may indicate a more corrected head posture. The mean gaze angle ranged between 10.3 and 13.1 degrees in these young children during relaxed standing, indicating small changes in the line of sight. The head on neck angle ranged from 25 to 31 degrees and indicates change in the position of the head relative to the neck. A decrease in this angle is considered to result in a 'poking chin' posture and may indicate stresses on the upper cervical spine. The lower limb angle gives an indication of the hip position (and therefore centre of gravity) over the base of support, as measured at the ankle. An increase in this angle may occur with increasing postural control related to positioning of the centre of gravity over the midfoot rather than the heel. Similar to the trunk angle, this lower limb angle uses landmarks a long distance apart, and thus our strict measurement protocols assisted in measurement accuracy.

### Effect of age on repeated posture measurements

The mean difference between repeated testing of any angle (as shown in Table 5) was less than three degrees. These figures need to be considered in relation to the 95% CI around the mean difference in each set of repeated measures. This data provides the first known information on 'usual' postural variability expected in children of these ages.

Table 5 illustrates the impact of age on postural performance during repeated testing. The lack of test – retest effect can be seen in all instances (all angles, all age groups), where the 95% confidence intervals span zero. As suggested however, by the low power for detecting differences in three of the repeated measures (neck, gaze and head on neck angles), further testing with larger numbers is required.

Multivariate ANOVA models confirmed that age had a significant influence at p < 0.01, on repeated testing for four of the angles, with only the gaze angle not being influenced by age. This finding adds support to the importance of stable gaze for orientation in children [1].

### Effect of age and other predictors on posture angles

The effect of age on measures of standing posture is clearly demonstrated in Table 3, which shows the step-wise decrease in the neck angle and the stepwise increase in head-on-neck angle as children get older. The changes in these two angles suggest taller, more corrected upright posture and may reflect growing postural maturity with age [14].

Figure 3 also demonstrates the low relative variability for the neck angle and the head-on-neck angles in each age group and the similarity of the variability across the age groups for these two angles. In comparison, there was high variability in general and quite different variability for each age group for the trunk angle and the lower limb angle. Excessive postural sway recognized in younger children, and measured in this study by movement of the greater trochanter over the base of support, may have influenced the variability demonstrated in these latter two angles [1]. Long levers were also associated with the trunk and lower limb angles. However, the comparative low variability of the neck angle, which also used a marker over the greater trochanter, may reflect a stable relationship between this hip point and markers in the head and neck region, in children of these ages. Under standardized positioning instructions, the normal high variability associated with movement of the eyes (as measured by the canthus, for determining the gaze angle), can be seen in the Figure 3.

It was considered that the measure of age in this sample of subjects may have reflected anthropometric growth (measured as height and weight) as well as developments in motor control influencing the body's ability to balance against the forces of gravity [26]. Age was found to be strongly associated with height (r^2 ^= 0.84) and moderately associated with weight and motor control (r^2 ^= 0.67, 0.56 respectively). These findings concur with current literature on paediatric development [21]. Neither pain reported on the day of testing, nor pain in the previous week, was significantly associated with age, or with differences between repeated measures of the angles.

However, as univariate predictors, motor control, height and weight were unconvincingly associated with four of the five posture angles (Table 4). The strongest findings were found for the trunk angle, where approximately 20% of the univariate associations were explained by motor control, height or weight. This association may be explained by the nature of this angle, which involves multi-segments of the body, using landmarks on C7 and the greater trochanter, and thus requires motor control between the head and trunk.

## Conclusion

On testing of repeatability of five postural angles in children 5–13 years, there was no significant effect of repeated testing. Increasing age influenced four of the five postural angles, with the only angle not affected by age being the gaze angle. Height, weight and motor control explained approximately 20% of the variability in the trunk angle, but explained very little of the variability in the other four angles. While the subject numbers in this study are small, the findings provide useful information on which further studies in posture and its development in pre-adolescent children can be based.

## Competing interests

The author(s) declare that they have no competing interests.

## Authors' contributions

KG conceived of the study, participated in the design of the study and conducted the statistical analysis.

MMc participated in the design of the study, collected the data and drafted the manuscript.

## Pre-publication history

The pre-publication history for this paper can be accessed here:



## References

[B1] Shumway-Cook A, Woollacott MH (2001). Motor control: theory and practical applications.

[B2] Straker L, Mekhora K (2000). An evaluation of visual dislay unit placement by electromyography, posture, discomfort and preference. International Journal of Industrial Ergonomics.

[B3] Steele E, Bialocerkowski A, Grimmer K (2003). The postural effects of load carriage on young people; a systematic review. BMC Musculoskeletal Disorders.

[B4] Kendall FP, McCreary EK (1983). Muscles testing and function.

[B5] Wickens JS, Kiputh OH (1937). Body mechanic analysis of Yale University freshmen. Research Quarterly.

[B6] Watson AWS, Mac Donncha C (2000). A reliable method for the assessment of posture. Journal of Sports Medicine and Physical Fitness.

[B7] Straker L, Jones KJ, Miller J (1997). A comparison of the posture assumed when using laptop computers and desktop computers. Applied Ergonomics.

[B8] Grimmer K, Dansie B, Milanese S, Pirunsan U, Trott P (2002). Adolescent standing postural response to backpack loads: a randomized controlled experimental study. BMC Musculoskeletal Disorders.

[B9] Bloomfield J, Ackland TR, Elliot BC (1994). Applied Biomechanics and Anatomy in Sport.

[B10] Karlin LI, Nicholas JA, Hershman EB (1986). Injury to the hip and pelvis in the skeletally immature athlete. The Lower Extremity and Spine in Sports Medicine.

[B11] Wojtys EM (1987). Sport injuries in the immature athlete. Orthopaedic Clinics of North America.

[B12] Forssberg H, Nashner LM (1982). Ontogenetic development of postural control in man: adaptation to altered support and visual conditions during stance. J Neuroscience.

[B13] Shumway-Cook A, Woollacott MH (1985). The growth of stability: postural control from a developmental perspective. Journal of Motor Behaviour.

[B14] Woollacott MH, Shumway-Cook A, Williams HG, Woollacott MH, Shumway-Cook A (1989). The development of posture and balance control in children. Development of Posture and gait Across the Life Span.

[B15] Grieve GP (1988). Common Vertebral Joint Problems.

[B16] Trott PH, Grant R (1994). Management of selected cervical syndromes. Physical Therapy of the Cervical and Thoracic Spine.

[B17] Trott PH, Grant R, Twomey LT, Taylor JR (2000). Manipulative physical therapy in the management of selected low lumbar syndromes. Physical Therapy of the Low Back.

[B18] Gandevia SC, Phegan CML (1999). Peripheral distortion of the human body image produced by local anaesthesia, pain and cutaneous stimulation. Journal of Physiology.

[B19] Grimmer K, Williams M, Gill T (1999). The association between adolescent head-on-neck posture, backpack weight, and anthropometric features. Spine.

[B20] Brace DK (1927). Measuring Motor Ability- A Scale of Motor Ability Tests.

[B21] Malina R, Bouchard C, Bar-Or O (2004). Growth, maturation, and physical activity.

[B22] Dawson B, Trapp R (2001). Basic and clinical biostatistics.

[B23] Anthony D (1999). Understanding advanced statistics: A guide for nurses and health care researchers.

[B24] PS-Power and Sample size calculation setup. http://www.mc.vanderbilt.edu/prevmed/ps/.

[B25] National Center for Health Statistics Database. http://www.cdc.gov/growyhcharts.

[B26] Usui N, Maekawa K, Hirasawa Y (1995). Development of the upright postural sway in children. Developmental Medicine and Neurology.

